# Author Correction: Circulating tumor DNA analysis depicts subclonal architecture and genomic evolution of small cell lung cancer

**DOI:** 10.1038/s41467-019-08570-x

**Published:** 2019-01-29

**Authors:** Jingying Nong, Yuhua Gong, Yanfang Guan, Xin Yi, Yuting Yi, Lianpeng Chang, Ling Yang, Jialin Lv, Zhirong Guo, Hongyan Jia, Yuxing Chu, Tao Liu, Ming Chen, Lauren Byers, Emily Roarty, Vincent K. Lam, Vassiliki A. Papadimitrakopoulou, Ignacio Wistuba, John V. Heymach, Bonnie Glisson, Zhongxing Liao, J. Jack Lee, P. Andrew Futreal, Shucai Zhang, Xuefeng Xia, Jianjun Zhang, Jinghui Wang

**Affiliations:** 1 Department of Medical Oncology, Beijing Chest Hospital, Capital Medical University, Beijing Tuberculosis and Thoracic Tumor Research Institute, 101149 Beijing, China; 2 Geneplus-Beijing, 102206 Beijing, China; 3 Geneplus-Beijing Institute, 102206 Beijing, China; 4 Beijing Key Laboratory for Drug Resistance Tuberculosis Research, Beijing Chest Hospital, Capital Medical University, Beijing Tuberculosis and Thoracic Tumor Research Institute, 101149 Beijing, China; 5 Department of Radiation Oncology, Zhejiang Cancer Hospital, 310022 Hangzhou, China; 6 Department of Thoracic/Head and Neck Medical Oncology, University of Texas MD Anderson Cancer Center, Houston, TX 77030 USA; 7 Department of Translational Molecular Pathology, University of Texas MD Anderson Cancer Center, Houston, TX 77030 USA; 8 Department of Radiation Oncology, University of Texas MD Anderson Cancer Center, Houston, TX 77030 USA; 9 Department of Biostatistics, University of Texas MD Anderson Cancer Center, Houston, TX 77030 USA; 10 Department of Genomic Medicine, University of Texas MD Anderson Cancer Center, Houston, TX 77030 USA; 11 Houston Methodist Research Institute, Houston, TX 77030 USA

Correction to: *Nature Communications*; 10.1038/s41467-018-05327-w; published online 06 August 2018

The original version of this Article contained an error in Fig. 2, in which the left *y*-axis labels ‘tDNA’ and ‘ctDNA’ were inadvertently inverted. This has been corrected in the PDF and HTML versions of the Article.

