# Supplementary material for: Circulating tumor DNA analysis depicts subclonal architecture and genomic evolution of small cell lung cancer
Source: Nat Commun. 2018 Aug 6;9:3114. doi: 10.1038/s41467-018-05327-w (PMC6079068; doi:10.1038/s41467-018-05327-w)
Supplement: Supplementary file 1 — Supplementary Information [file 41467_2018_5327_MOESM1_ESM.pdf]

Circulating tumor DNA analysis depicts subclonal architecture and genomic evolution of small cell lung cancer

Supplementary Information

Nong et al.

Supplementary Figures

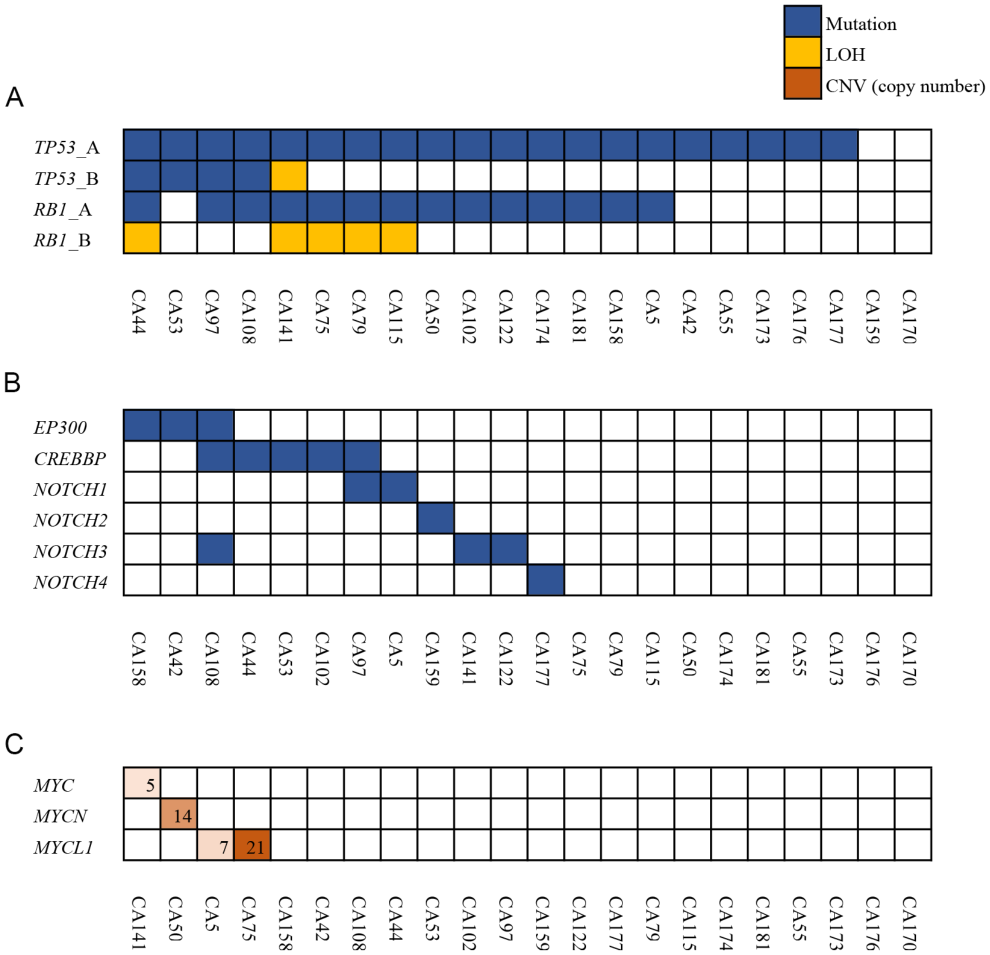

Supplementary Fig. 1. Genomic aberrations of previously reported frequently mutated cancer genes in SCLC detected in pretreatment ctDNA samples from 22 SCLC patients

(A) Mutations and LOH of *TP53* and *RB1*. Mutations (Blue) and LOH (Yellow) in both A and B allele of TP53 and RB1 gene were shown. (B) Mutations in *EP300*, *CREBBP* and NOTCH family members. (C) Copy number variations (CNV) in *MYC*, *MYCN* and *MYCL1* were detected in 4 samples.

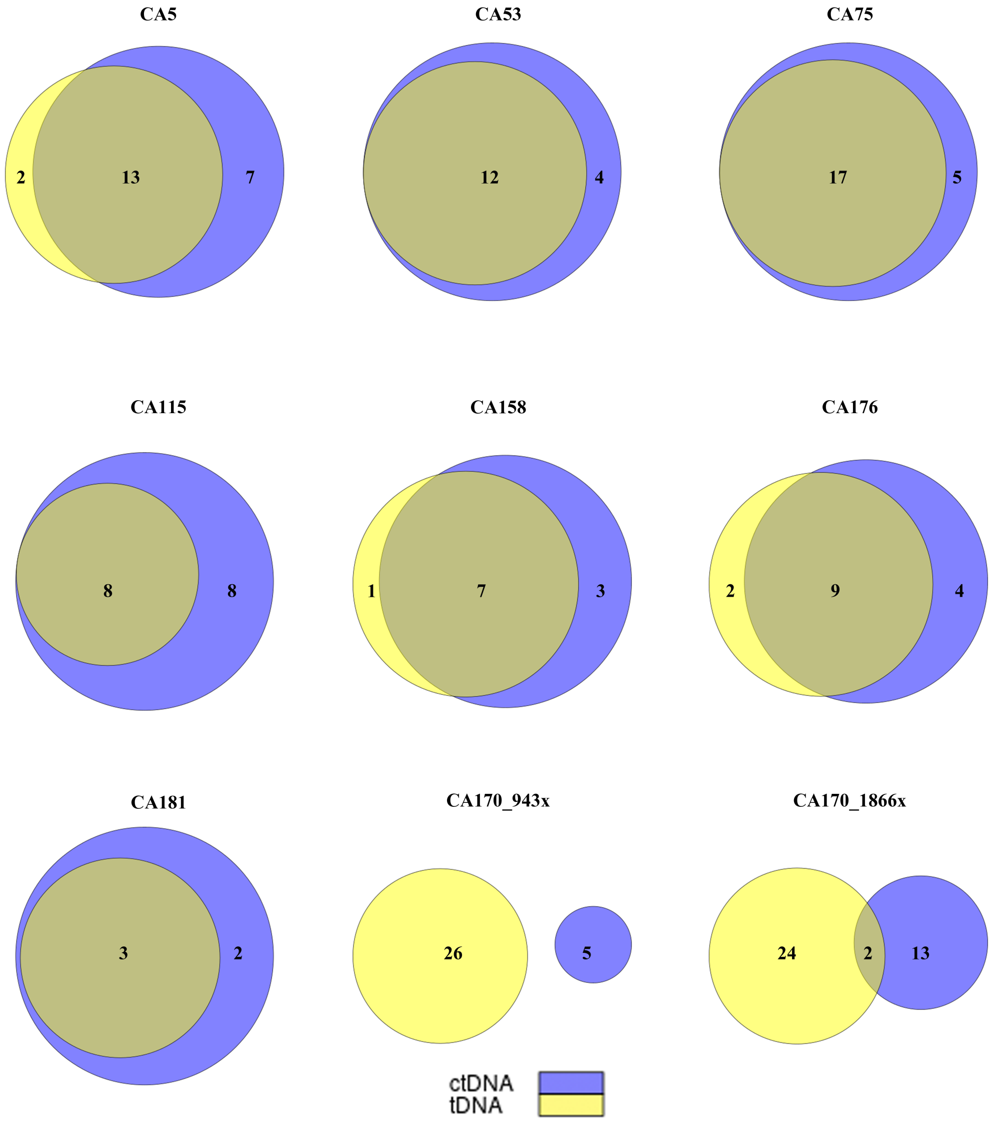

Supplementary Fig. 2. Shared and unique mutations in pretreatment ctDNA and paired pretreatment tumor samples in eight patients.

tDNA: tumor DNA. CA170_943x: sequencing depth of ctDNA at 943x.

CA170_1866x: sequencing depth of ctDNA at 1866x.

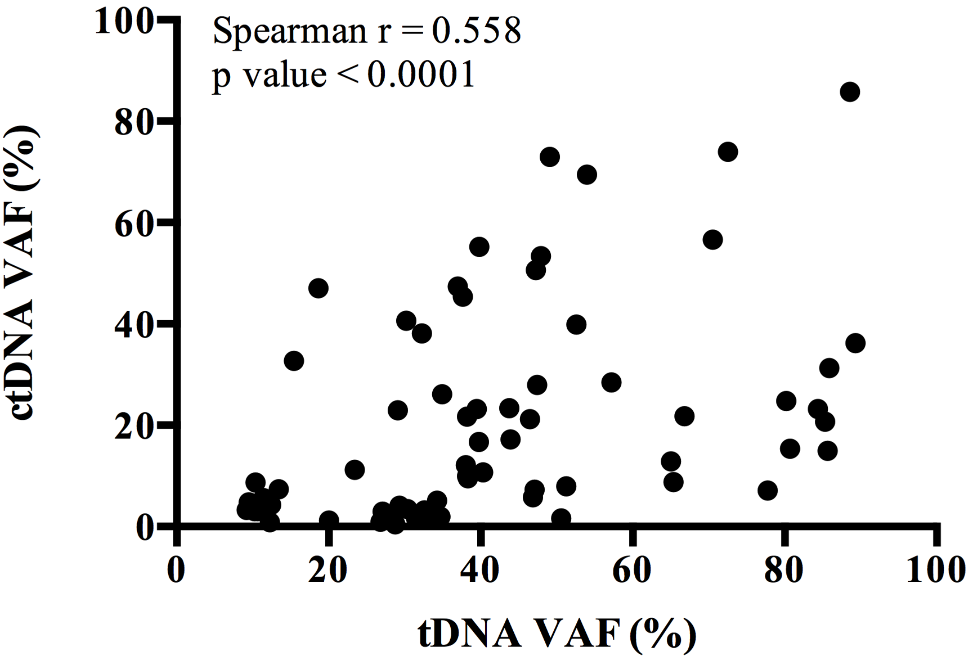

Supplementary Fig. 3. Correlation of shared mutation VAFs in ctDNA and tDNA.

VAFs of 69 shared mutations in paired ctDNA and tDNA samples were depicted and correlation is shown.

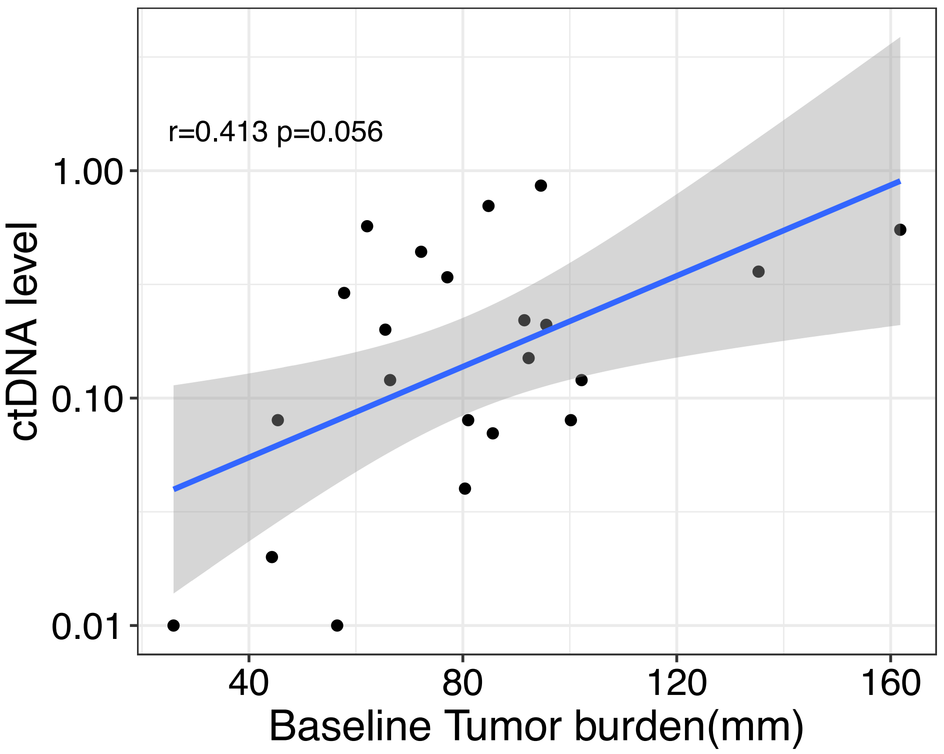

Supplementary Fig. 4. Correlation of baseline tumor burden and ctDNA levels.

ctDNA levels and tumor burden (sum of the longest diameters of the target lesions on CT scans) of 22 patients were measured before any treatment, and Spearman correlation analysis was done.

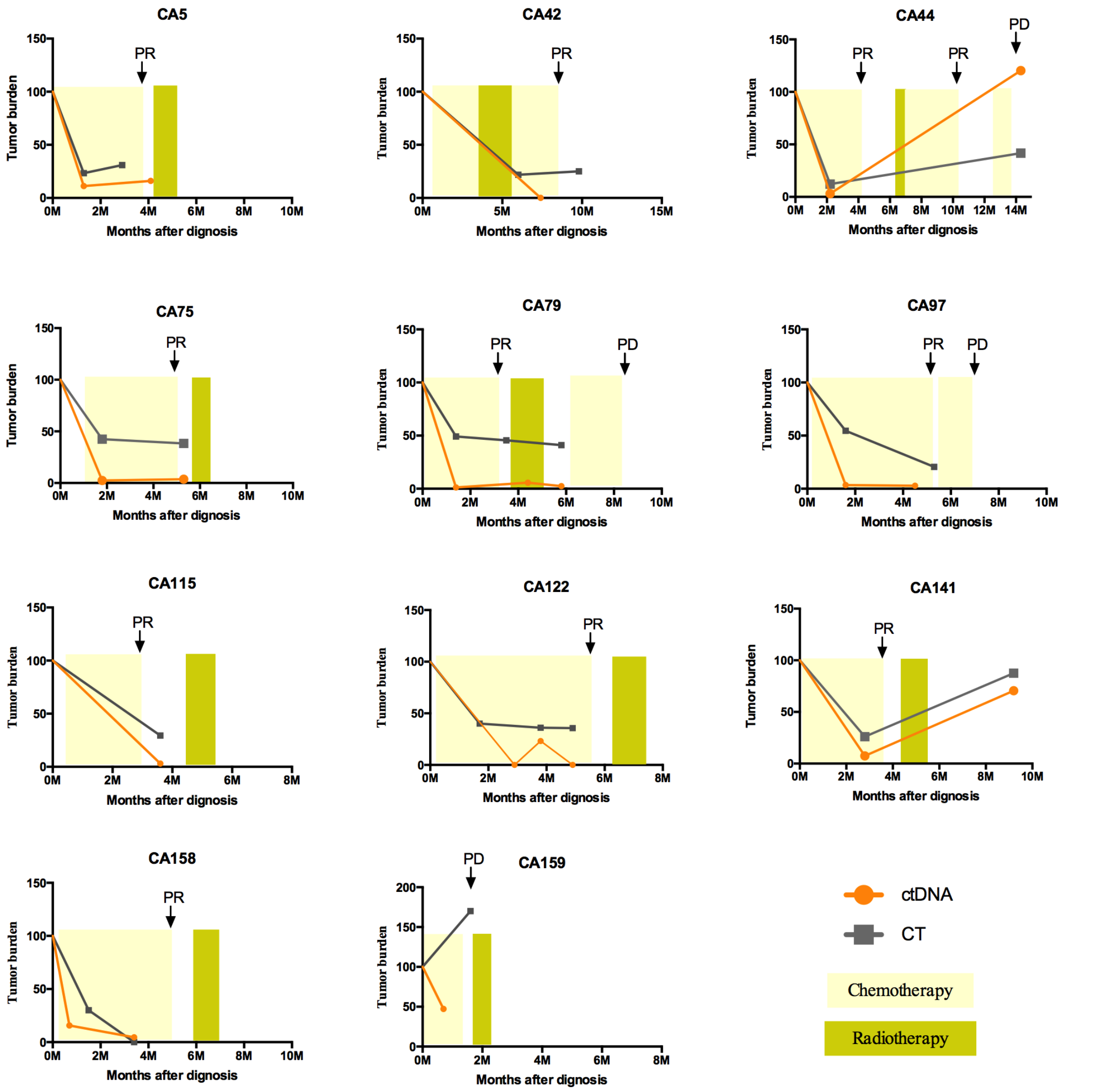

Supplementary Fig. 5. Dynamic changes of ctDNA level and measurable tumor lesions evaluated by CT scans in 11 patients with post-treatment ctDNA samples available.

Changes of ctDNA level and sum of longest diameters of target lesions measured by CT are shown. Chemotherapy or radiotherapy was indicated by yellow and green shadow, respectively. Patients response to therapy (PR: partial response; PD: progressive disease) are indicated by arrows.

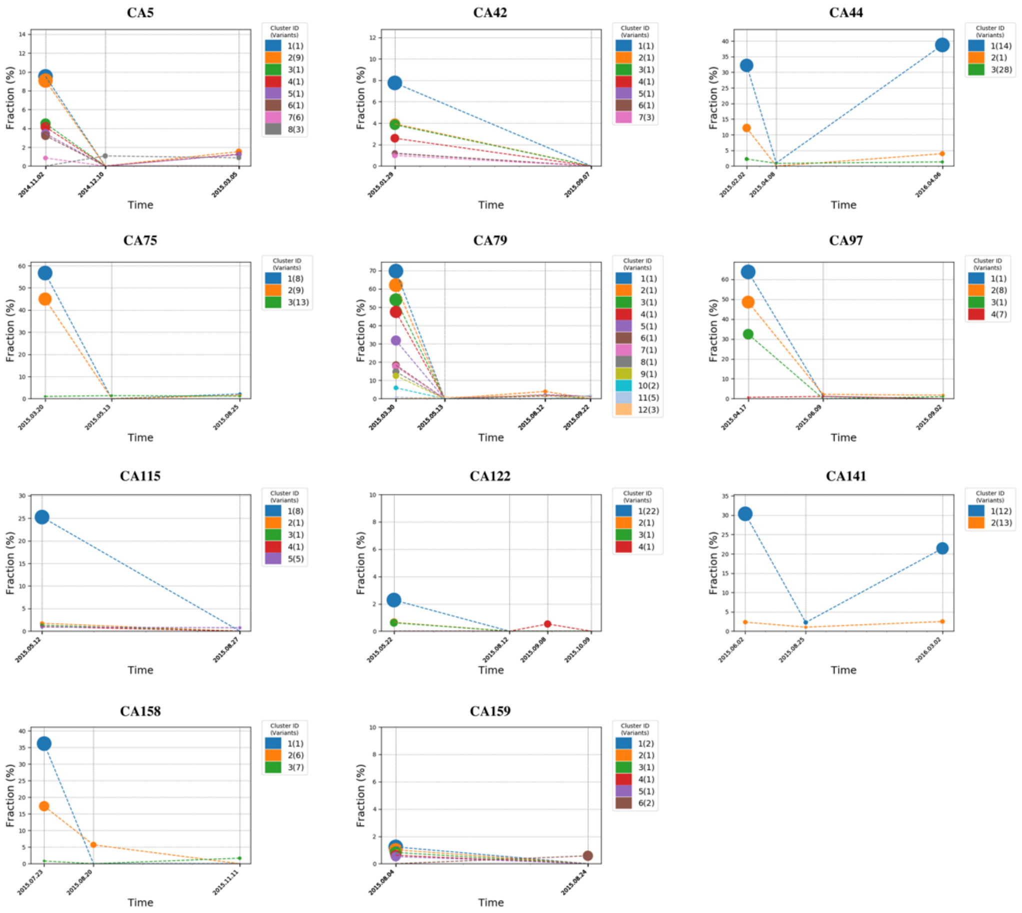

Supplementary Fig. 6. Dynamic changes of mutation clusters during treatment in the 11 patients with post-treatment ctDNA samples available.

Different mutation clusters were colored and ordered according to their relative abundance.
