# Supplementary material for: Circulating tumor DNA analysis depicts subclonal architecture and genomic evolution of small cell lung cancer
Source: Nat Commun. 2018 Aug 6;9:3114. doi: 10.1038/s41467-018-05327-w (PMC6079068; doi:10.1038/s41467-018-05327-w)
Supplement: Supplementary file 3 — Description of Additional Supplementary Files [file 41467_2018_5327_MOESM3_ESM.pdf]

## Description of Additional Supplementary Files

File Name: Supplementary Data 1
Description: Patient characteristics

File Name: Supplementary Data 2
Description: Gene list

File Name: Supplementary Data 3
Description: Somatic mutations detected in pre-treatment ctDNA in patients with SCLC

File Name: Supplementary Data 4
Description: Tumor DNA mutations in patients with SCLC

File Name: Supplementary Data 5
Description: Clinicopathologic and molecular factors and their effects on Progression-FreeSurvival and Overall-Survival by multivariate Cox proportional hazards regression analysis

File Name: Supplementary Data 6
Description: Dynamic changes of mutations in ctDNA

File Name: Supplementary Data 7
Description: Potential functional mutations specific to post-therapy ctDNA
